# Numerical Investigation of Fabricated MWCNTs/Polystyrene Nanofibrous Membrane for DCMD

**DOI:** 10.3390/polym13010160

**Published:** 2021-01-04

**Authors:** Asmaa Elrasheedy, Mohammed Rabie, Ahmed El-Shazly, Mohamed Bassyouni, S.M.S. Abdel-Hamid, Marwa F. El Kady

**Affiliations:** 1 Chemical and Petrochemicals Engineering Department, Egypt-Japan University of Science and Technology (E-JUST), Alexandria 21934, Egypt; mohammed.rabie@ejust.edu.eg (M.R.); elshazly_a@yahoo.com (A.E.-S.); marwa.elkady@ejust.edu.eg (M.F.E.K.); 2 Department of Chemical Engineering, Faculty of Engineering, Port Said University, Port Said 42526, Egypt; 3 Mechanical Power Engineering, Mansoura University, El-Mansoura 35516, Egypt; 4 Chemical Engineering Department, Faculty of Engineering Department, Alexandria University, Alexandria 21544, Egypt; 5 Materials Science Program, Zewail University of Science and Technology, City of Science and Technology, October Gardens, 6th of October, Giza 12578, Egypt; 6 Department of Chemical Engineering, the Egyptian Academy for Engineering and Advanced Technology, Affiliated to Ministry of Military Production, Al Salam City 3056, Egypt; shereenahmed@eaeat.edu.eg; 7 Polymeric Materials Research Department, City of Scientific Research and Technological Applications (SRTA-City), Borg El-Arab City, Alexandria 21934, Egypt

**Keywords:** MD, membrane distillation, composite membrane, numerical investigation, CFD, DCMD

## Abstract

The effect of compositing multiwalled carbon nanotubes (MWCNTs) with polystyrene (PS) to fabricate nanofibrous membrane by electrospinning technique and comparing the direct contact membrane distillation (DCMD) performance of the blank and composite membranes is evaluated numerically. Surface morphology of both the pristine and the composite membrane was studied by SEM imaging while the average fiber diameter and average pore size were measured using ImageJ software. Static water contact angle and porosities were also determined for both membranes. Results showed significant enhancement in both the hydrophobicity and porosity of the composite membrane by increasing the static water contact angle from 145.4° for the pristine PS membrane to 155° for the PS/MWCNTs composite membrane while the porosity was increased by 28%. Simulation results showed that at any given feed inlet temperature, the PS/MWCNTs membrane have higher permeate flux and better overall system performance.

## 1. Introduction

Water shortage crisis is now becoming more alarming concern due to the rapid increase in world population, industrialization, and limited freshwater resources [1,2,3,4,5]. United Nations as well as world health organization reported that over 50% of the countries worldwide will suffer from water shortage problems while millions of people already suffered or are suffering from fatal health issues due to contaminated water [6]. Hence, the need for alternative sources for fresh water rather than the conventional sources has pretty much gained the attention of scientists in the recent decades. Membrane technologies have proved their superiority over other conventional desalination techniques as they are more energy efficient, provide high quality of produced water, have high productivity, have effective separation performance, have low chemicals demand, and are cost effective [7,8,9].

Membrane distillation (MD), in particular, have some exclusive advantages over other membrane purification technologies in that the removal of nonvolatile contaminants is 100%, lower operating pressure than other pressure-dependent membrane separation techniques, and lower operating temperature than other thermal desalination technologies. MD is a thermally driven membrane separation technique in which only vapor molecules transfer through a highly porous, thermally stable, hydrophobic membrane due to the vapor pressure difference that is initiated originally from temperature difference on both sides of the membrane [10,11,12,13,14].

There are four main categories in MD: (a) direct contact membrane distillation (DCMD) where the hot feed stream and the cold permeate stream are in contact with the membrane directly. Vapors transfers from the feed side and condenses directly in the permeate side. (b) Air-Gap MD (AGMD) utilizes an air gap at the permeate side between the membrane and a thermally conductive condensation surface on which vapors transmitted from the feed side condenses [15]. (c) Sweeping gas MD (SGMD), where vapors transmitted from the feed side is swept by an inert gas to a condenser outside the module to be collected. (d) Vacuum membrane distillation (VMD), here, vacuum is initiated at the permeate side to collect all the vapors and decrease the losses by means of a vacuum pump. The vapors are collected in an external condenser outside the membrane module [15,16]. Among the previous configurations of MD, DCMD is the most widely studied, due its simple operation and equipment [17].

The main reason preventing the commercial spread of MD is the very specific MD membrane characteristics and low productivity if compared to other membrane purification techniques [10,18,19]. One of the key parameters governing the MD process is the intrinsic characteristics of the MD membrane, which should be highly hydrophobic, highly porous, and thermally stable [10,20]. Polystyrene (PS) is a hydrophobic, commercially available, cheap polymer and proved its good spinnability giving highly porous, hydrophobic mats with wide range of applications [21]. However, polymeric membranes, in general, have low fouling and scaling resistance and are less chemically and mechanically stable [22].

Recent studies are focused on enhancing membranes characteristics by adding fillers to the polymer matrix to produce membranes with predesigned properties. Utilization of carbon nanotubes (CNTs) as membrane fillers have proved to enhance the performance of membranes, by enhancing their mechanical properties, and decrease the fouling tendency that is usually encountered in hydrophobic membranes due to the antimicrobial property of CNTs [22,23]. There are three main methods to incorporate CNTs within polymer matrices that is used in MD, namely, CNTs self-supporting bucky-paper, CNTs-immobilized membrane, and CNTs blended membranes [24,25,26]. However the later method proved to be most efficient to improve the membranes performance and mechanical intensity compared to the other two techniques and the simplest among them too [27]. CNTs are also expected to add some promising characteristics to MD membranes by enhancing membrane hydrophobicity, pore size, and porosity due to their hollow and nanosized structure, hydrophobic nature, and durability [28].

Another key factor controlling the properties of produced membranes is the membrane fabrication technique itself. Electrospinning technique proved to produce nanofibrous membranes from PS and other polymeric materials with controlled fiber diameters and morphology, high porosity, and relatively high hydrophobicity making it a very good candidate for MD application [21,29,30,31]. Electroblowing technique was also reported for fabrication of hydrophobic nanofibrous mats from PS for DCMD [32,33,34,35,36,37]. Table 1 presents PS membrane systems reported in literature for DCMD application for water desalination.

According to best of our knowledge, no previous studies investigated PS/MWCNTs composite membrane for the application of direct contact membrane distillation (DCMD). The present work aims to fabricate a novel membrane by adding multiwalled carbon nanotubes (MWCNTs) to PS membrane to enhance its properties and increase the productivity. After characterization of the new fabricated membrane, its performance has been compared numerically with the pristine PS membrane using a commercial software (Ansys 2019R3) for the application on a DCMD system.

## 2. Materials and Methods

Polystyrene (PS, M_w_ = 260,000) in pellets form were purchased from ACROS organics, Morris Plains, NJ, USA. *N*,*N*-dimethyl formamide (DMF) for analysis was purchased from ACS, ISO was purchased from Merk KGaA, Darmstadt, Germany. Multi-walled Co., and multiwalled carbon nanotubes (MWCNTs, D×L 110–170 nm × 5–9 µm, purity ≥90% carbon basis) %) were purchased from Sigma AldrichChemie GmbH TAUFKIRCHEN, Germany and used as it is.

### 2.1. Membrane Fabrication

The polymer solution was prepared by dissolving PS pellets in DMF and stirred for 6 h at room temperature to obtain 18 wt% polymer solution. The electrospinning conditions were as follows: solution delivery rate was fixed at 1 mL/h, tip to collector distance was maintained at 15 cm, and the applied potential difference was 30 kV. The composite membrane was prepared by adding 2 wt% of MWCNTs to the polymer solution after complete dissolution and stirred for 1 h followed by sonication for 1 h. The electrospinning setup used was NANON-01A (MECC CO., Ltd., Fukoka, Japan). Fabricated membranes were then left in oven at 60 °C overnight to get rid of any residual solvents, and then, they were cold-pressed to ensure membrane integrity at 2 MPa for 1 min.

### 2.2. Membrane Characterization

#### 2.2.1. Scanning Electron Microscope (SEM)

Morphologies of CNTs and blank and composite membrane were investigated using SEM (JCM-6000PLUS NeoScope Benchtop SEM, Tokyo, Japan). The samples were fixed on a carbon tape and mounted on an aluminum stub. Bio-Rad SEM coating system was used, and the samples were put under vacuum for 2 min at 20 mV accelerating voltage.

#### 2.2.2. Single Drop Contact Angle

A drop size analyzer system (DSA100, KRÜSS, Hamburg, Germany–with image analysis) was used to examine the water contact angle for the neat as well as the composite membrane to determine their degree of hydrophobicity. Measurements were carried out at 10 different spots per membrane, and the average value was reported.

#### 2.2.3. Thickness and Porosity

The thickness of the membranes was measured using a digital micrometer in 10 different spots, and the average was taken. Porosities of the membranes were estimated using gravimetric method [29] using the following equation:

(1) $$\varepsilon =\;\frac{\left(W_{w}-W_{d}\right)/\rho_{i}}{\left(W_{w}-W_{d}\right)/\rho_{i}+W_{PS}/\rho_{PS}}$$

where $W_{w}$, $W_{d}$ and $W_{PS}$ are the wet membrane weight, the dry membrane weight and the PS weight, respectively; $\rho_{i}$ and $\rho_{PS}$ are the densities of the isopropyl alcohol and polystyrene densities respectively. Membrane porosities were evaluated after cold-pressing.

#### 2.2.4. Pore Sizes and Fiber Diameter

The average pore size and the fiber diameter of the blank and composite were estimated using ImageJ software (LOCI, University of Wisconsin, Madison, WI, USA).

#### 2.2.5. Fourier-Transform Infra-Red (FTIR)

Samples of MWCNTs, PS, and PS/MWCNTs neat and composite nanofibrous membranes were blended in ratio of 1:100 w/w with KBr to form pellets. Then, the sample pellets were studied using FTIR (Vertex 70, Bruker scientific instruments, Baden-Württemberg, Germany) at ambient conditions with wave range of 4000–400 cm^−1^.

#### 2.2.6. Numerical Analysis

A three-dimensional model was drawn and discretized to be solved using a commercial software package (Ansys 2019 R3, Canonsburg, PA, USA). At first, the system was tested for mesh dependence starting from coarse mesh to a fine one until the solution becomes mesh independent. Then, the suitable boundary conditions were set on the system boundaries to be velocity inlet at both inlet ports and pressure outlets at feed and permeate outlet sections. The software uses the finite volume method to solve the governing equations, and the convergence criteria were set to be at least 1 × 10^−9^ for all equations. The system is governed by the mass, momentum and energy equations in steady three-dimension form. Moreover, the solution is subjected to the following assumptions:

- Steady and laminar flow and incompressible fluid.
- All properties (fluid and material) are constant within the operating range.
- No heat losses from the system to the surrounding.

##### Model Discerption

The analytical model was performed on a typical dimension as the experimental model. The model consisted of three layers, i.e., the first layer was the feed channel in which hot saltwater flowed, while the second layer was the hydrophobic porous membrane which was followed by the permeate channel within which the pure cold-water flowed. The three layers had identical surface area, which had an equal length and width of 50 mm. The height of the feed and permeate channels were constant, 2 mm, while the height of the membrane (thickness) was 500 ± 4 μm.

##### Governing Equations

The control volume is subjected to the following governing equations:

Continuity:

(2) $$\frac{\partial \rho U}{\partial x}+\frac{\partial \rho V}{\partial y}+\frac{\partial \rho W}{\partial z}=M$$

X–Momentum equation:

(3) $$U\frac{\partial \rho U}{\partial x}+V\frac{\partial \rho U}{\partial y}+W\frac{\partial \rho U}{\partial z}=-\frac{\partial p}{\partial x}+\mu \left(\frac{\partial^{2}U}{\partial x^{2}}+\frac{\partial^{2}U}{\partial y^{2}}+\frac{\partial^{2}U}{\partial z^{2}}\right)$$

y–Momentum equation:

(4) $$U\frac{\partial \rho V}{\partial x}+V\frac{\partial \rho V}{\partial y}+W\frac{\partial \rho V}{\partial z}=-\frac{\partial p}{\partial y}+\mu \left(\frac{\partial^{2}V}{\partial x^{2}}+\frac{\partial^{2}V}{\partial y^{2}}+\frac{\partial^{2}V}{\partial z^{2}}\right)$$

z–Momentum equation:

(5) $$U\frac{\partial \rho W}{\partial x}+V\frac{\partial \rho W}{\partial y}+W\frac{\partial \rho W}{\partial z}=-\frac{\partial p}{\partial z}+\mu \left(\frac{\partial^{2}W}{\partial x^{2}}+\frac{\partial^{2}W}{\partial y^{2}}+\frac{\partial^{2}W}{\partial z^{2}}\right)$$

Energy equation:

(6) $$U\frac{\partial {\rho C}_{p}T}{\partial x}+V\frac{\partial {\rho C}_{p}T}{\partial y}+W\frac{\partial {\rho C}_{p}T}{\partial z}=k\left(\frac{\partial^{2}T}{\partial x^{2}}+\frac{\partial^{2}T}{\partial y^{2}}+\frac{\partial^{2}T}{\partial z^{2}}\right)+H$$

where U, V and W are the velocity components in x, y and z directions, respectively; ρ, μ, $C_{p}$, p, and T are the fluid density, viscosity, specific heat, pressure, and temperature, respectively; while M and H are the mass source and heat source, respectively.

##### System Metrics

In this research, we mainly studied the effect of operating and design parameters on the performance of PS/MWCNTs composite membrane and compared it with the blank membrane. The studied metrics for evaluating the performance were the system permeate flux (J), system thermal efficiency (η), and temperature polarization coefficient (TPC) ϕ. These metrics could be expressed as the following equations:

(7) $$J=\Psi \left(P_{\mathrm{vf}}-P_{\mathrm{vp}}\right)$$

where Ψ is the mass transfer coefficient based on Knudsen molecular diffusion model, while $P_{\mathrm{vf}}$ and $P_{\mathrm{vp}}$ are the vapor pressure difference on both sides of the membrane.

(8) $$\eta =\frac{Q_{v}}{Q_{v}+Q_{cond}}$$

where $Q_{v}$ and $Q_{cond}$ are the amount of heat transferred with vapor (useful) and heat conduction through the membrane (losses), respectively.

(9) $$\phi =\frac{T_{mf}-T_{mp}}{T_{bf}-T_{bp}}$$

$T_{mf}$ and $T_{mp}$ are the membrane/feed and membrane/permeate interface temperatures, respectively; while $T_{bf}$ and $T_{bp}$ are the bulk temperatures at the feed and permeate sides, respectively.

## 3. Results

**Figure 1:**
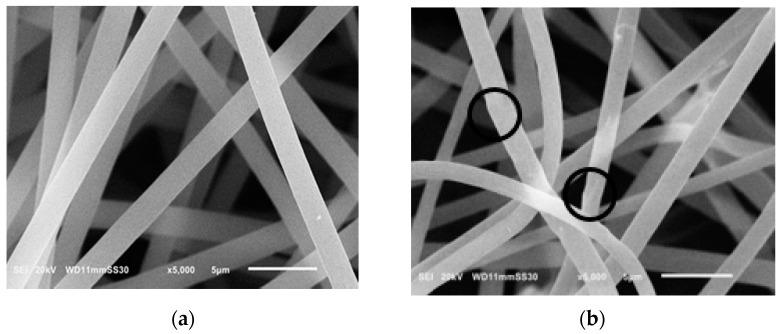
SEM images of (**a**) polystyrene (PS) blank membrane and (**b**) PS/multiwalled carbon nanotubes (MWCNTs) composite membranes.

**Figure 2:**
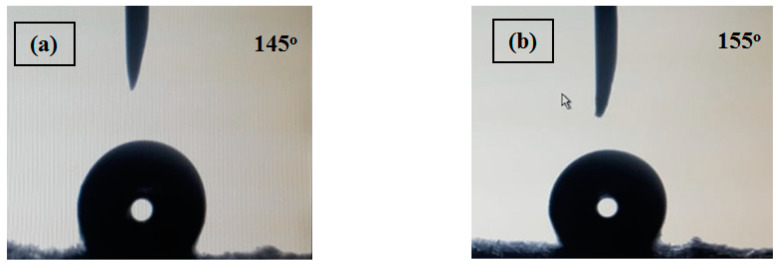
Contact angle of (**a**) PS and (**b**) PS/MWCNTs membranes.

Figure 1 presents the SEM imaging of the electrospun pristine PS and PS/MWCNTs composite membranes. The Bead-free, smooth, and uniform morphology of the PS membrane observed in Figure 1a indicates that the electrospinning conditions was suitable for the prepared polymer solution concentration yielding a continuous mat of fibers with average fiber diameter of 1.783 µm and average pore size of 0.423 µm. This stratified structure kind yielded a membrane surface with high roughness with contact angle of 145.4° as could be observed in Figure 2a.

PS/MWCNTs composite membrane SEM images are presented in Figure 1b showed that the average fiber diameter decreased to 1.545 µm, while the average pore size was found to decrease to 0.357. This may be attributed to the increased conductivity of the solution due to the presence of MWCNTS. Presence of the MWCNTs in the composite membrane also enhanced the contact angle by increasing it to 155° (Figure 2b) as a result of the increased surface roughness [34]. Addition of MWCNTs was also found to increase the porosity by approximately 28%; this increase in membrane porosity with incorporation of MWCNTs in the polymer matrix is in agreement with others reported in literature due to the nanos and hollow structure of the MWCNTS [4,35,36,37]. The porosity and hydrophobicity of the fabricated composite membrane PS/MWCNTs is higher than that reported by others for PS membranes in DCMD [21,29,38]. Table 2 summarizes the main membrane characteristics for the neat and the composite membrane.

**Figure 3:**
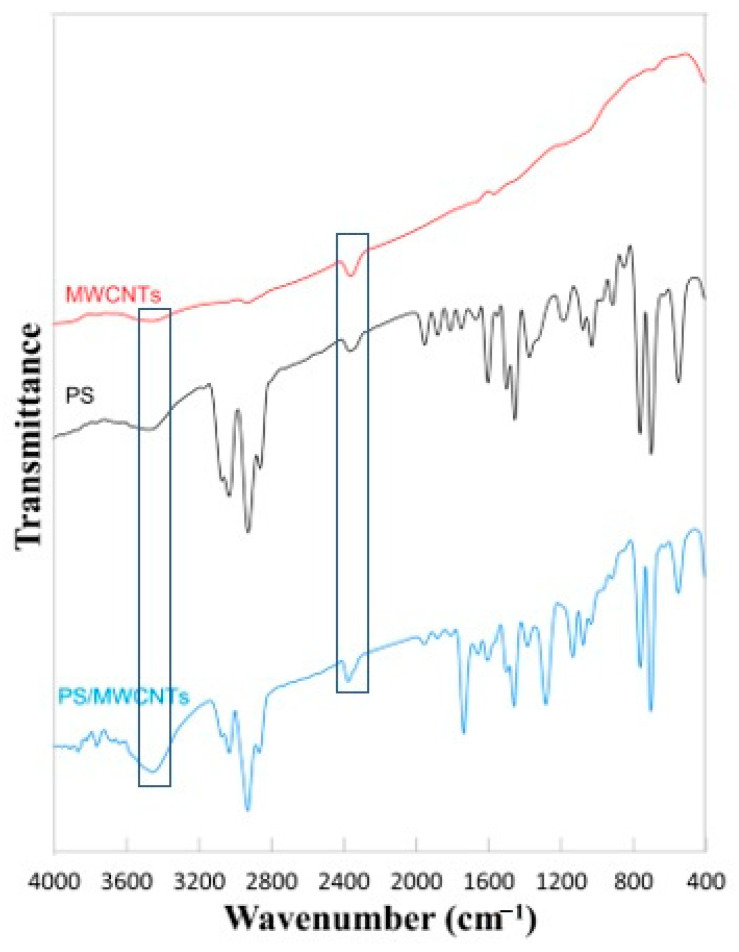
FTIR spectra of MWCNTs and PS neat and PS/MWCNTs composite and nanofibrous membranes.

The FTIR spectra of MWCNTs, PS, and PS/MWCNTs neat and composite nanofibrous membranes are presented in Figure 3. MWCNTs show some weak peaks that corresponds to OH groups due to the absorption of some water molecules and C=O that is due to oxidation of carbon chains. These later peaks can be observed at 2355.16 and 2925.15 cm^−1^, respectively [39]. However, the weak peak observed at 1640.51 cm^−1^ in the IR spectrum of MWCNTs is related to the stretching of the MWCNTs backbone [40]. PS characteristic peaks are reported at 3026.41, 2921.29, 2854.74, 1945.28, 1742.74, 1446.66, 1491.99, 1071.49, 1023.27, 756, 694.4, and 542.98 cm^−1^. The C–H symmetric and asymmetric vibrations are observed at 3026.41, 2921.29, and 2854.74 cm^−1^. Further, 1945.28 and 1742.74 cm^−1^ are attributed to the aromatic monosubstitution and weak aromatic overtone. The peaks at 1446.66 and 1491.99 cm^−1^ are assigned to the bending vibrations of CH_2_. The 1071.49 and 1023.27 cm^−1^ peaks are assigned to in plane flexion of C–H in the plane. The out-of-plane bending of the phenyl ring is observed at 756 cm^−1^. The phenyl ring out-of-plane deformation peak is observed at 540 cm^−1^ [34,41,42]. Addition of MWCNTs strengthen the characteristic peaks of PS as can be seen in the PS IR spectrum [43].

**Figure 4:**
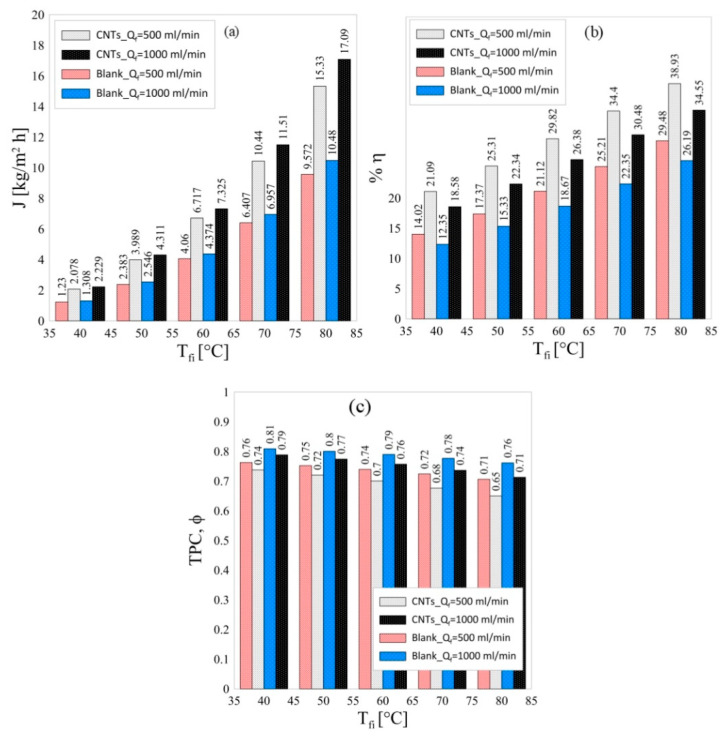
Effect of feed inlet temperature on (**a**) the system permeate flux (J), (**b**) system thermal efficiency (η), and (**c**) temperature polarization coefficient (ϕ) at two different flow rates and for blank and composite membranes.

Figure 4 shows the effect of the feed inlet temperature on the performance of the blank and fabricated membranes at two different feed flow rates. As could be seen in Figure 4a at any given feed inlet temperature, increasing the feed flow rate increases the membrane flux (produced pure water) substantially. Moreover, the flux obtained from PS/MWCNTs composite membrane is much higher than that of PS membrane at any given feed inlet temperature which is attributed to the higher porosity of the composite membrane that facilitates the passage of the vapor molecules through the membrane and at any given temperature.

Although increasing the feed inlet temperature have a significant effect on increasing the permeate flux obtained from MD [44], the feed inlet temperature in systems using PS cannot exceed 75 °C as the glass transition temperature of PS is between 80 and 100 °C [19]. However, other studies reported that incorporation of MWCNTs can shift the glass transition temperature of PS to higher values in the range of 8–20 °C [45,46]. This increase in the glass transition temperature of PS filled with MWCNTs may be attributed to the reduction in the polymer chains mobility due to the high interaction between the CNTs and PS [46]. This increase in the glass transition temperature of the composite membrane PS/MWCNTs will enable the operation of the DCMD system at higher temperatures (the feed inlet temperature) for the actual application of the DCMD setup. Hence, the obtained permeate flux can be enhanced significantly.

On the other hand, it can be observed from Figure 4b that the thermal efficiency increases with increasing the feed inlet temperature. It also shows that the composite membrane has higher efficiency than that of the blank membrane at any given feed inlet temperature and that could be explained as a consequence of the increased membrane flux, which increases the amount of useful heat $Q_{v}$ and hence increases the thermal efficiency according to Equation (8). Figure 4c shows that the composite membrane has a slightly lower TPC than that of the blank membrane at all operating conditions. This could be explained as the increased flux for the composite membrane needs more heat for vaporization, so it decreases the feed/membrane interface temperature and thus, deviated from the feed bulk temperature.

In general, increasing the feed inlet temperature increases the resultant flux and enhances the overall efficiency as a result of the increased vaporization of feed water at higher temperatures [47]. Furthermore, the PS/MWCNTs has much higher improved performance than that of the blank membrane.

**Figure 5:**
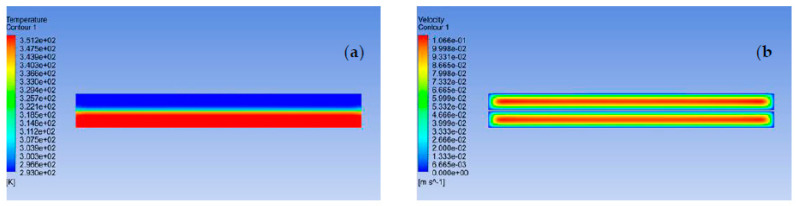
(**a**) Temperature and (**b**) velocity contours of DCMD numerical model of the PS/MWCNTs composite membrane running at 20 and 80 °C inlet temperatures of permeate and feed channels, respectively, and porosity of 0.72.

The velocity and temperature contours of the PS/MWCNTs composite membrane are presented in Figure 5. The velocity and temperature contours were evaluated midway in the feed and permeate channels normal to the flow direction at 20 and 80 °C permeate and feed temperatures, respectively, and flow rate of 500 mL/min.

## 4. Conclusions

In the present work, effect of addition of multiwalled carbon nanotubes (MWCNTs) to polystyrene (PS) nanofibrous membrane is studied, and its performance was evaluated numerically on DCMD cell. PS/MWCNTs composite membrane showed superior properties and performance if compared to the pristine PS membrane. Adding MWCNTs to PS enhanced the hydrophobicity of the membrane by increasing the contact angle from 145.4° to 155° and increasing the porosity by 28%. Numerical investigation showed that at any given inlet feed temperature, the composite PS/MWCNTs membrane showed superior performance and overall system efficiency if compared to the pristine PS membrane. Further investigation of the fabricated membranes should be carried out experimentally for validation of the simulation data.

## Figures and Tables

**Table 1:** Different polystyrene (PS) membrane systems for water desalination via direct contact membrane distillation (DCMD).

Parameter	[19]	[22]	[31]	[37]
Feed flowrate (L/min)	0.6	0.6	0.24–0.6	0.054
Permeate flowrate (L/min)	0.6	0.6	0.24–0.6	0.054
Difference in operating temperature between the feed and the permeate (°C)	50	63	25–60	40
Contact angle	150.2 ± 1.2°	114 ± 1°	154.52°	119.6°
Membrane thickness (μm)	60	147 ± 4	65	–
Porosity (%)	69	84	74.65	–
Feed solution concentration (ppm)	35,000	35,000	35,000	7000

**Table 2:** Characteristic properties of the pristine and composite membranes.

Membrane	Porosity	Contact Angle	Avg Fiber Diameter	Pore Size
PS	0.56	145.4°	1.783 µm	0.423 µm
PS/MWCNTs	0.72	155	1.545 µm	0.357 µm

## Data Availability

Not applicable.

## References

[1] Takeuchi K., Takizawa Y., Kitazawa H., Fujii M., Hosaka K., Ortiz-Medina J., Morelos-Gomez A., Cruz-Silva R., Fujishige M., Akuzawa N. (2018). Salt rejection behavior of carbon nanotube-polyamide nanocomposite reverse osmosis membranes in several salt solutions. Desalination.

[2] El-Mehalmey W.A., Safwat Y., Bassyouni M., Alkordi M.H. (2020). Strong Interplay between Polymer Surface Charge and MOF Cage Chemistry in Mixed-Matrix Membrane for Water Treatment Applications. ACS Appl. Mater. Interfaces.

[3] Wang L., Wang Y., Wu L., Wei G. (2020). Fabrication, Properties, Performances, and Separation Application of Polymeric Pervaporation Membranes: A Review. Polymers.

[4] Bassyouni M., Mansi A.E., Elgabry A., Ibrahim B.A., Kassem O.A., Alhebeshy R. (2019). Utilization of carbon nanotubes in removal of heavy metals from wastewater: A review of the CNTs’ potential and current challenges. Appl. Phys. A.

[5] Gutub S.A., Bassyouni M., Abdel-Hamid S.M.S. (2013). Dissolved solids adsorption of freshwater using synthesized bio-foam composite. Life Sci. J..

[6] Zhang Z., Zhang W., Lichtfouse E. (2020). Membranes for Environmental Applications.

[7] Elrasheedy A., Nady N., Bassyouni M., El-Shazly A.H. (2019). Metal Organic Framework Based Polymer Mixed Matrix Membranes: Review on Applications in Water Purification. Membranes.

[8] Wang R., Chen D., Wang Q., Ying Y., Gao W., Xie L. (2020). Recent Advances in Applications of Carbon Nanotubes for Desalination: A Review. Nanomaterials.

[9] Ali S., Rehman S.A.U., Luan H.-Y., Farid M.U., Huang H. (2019). Challenges and opportunities in functional carbon nanotubes for membrane-based water treatment and desalination. Sci. Total Environ..

[10] Camacho L.M., Dumée L.F., Zhang J., Li J.-D., Duke M., Gomez J.D., Gray S. (2013). Advances in Membrane Distillation for Water Desalination and Purification Applications. Water.

[11] Alanezi A.A., Elhenawy Y., Goodarzi M., Safaei M.R. (2020). The Effect of Inclination Angle and Reynolds Number on the Performance of a Direct Contact Membrane Distillation (DCMD) Process. Energies.

[12] Elminshawy N.A., Gadalla M.A., Bassyouni M., El-Nahhas K., Elminshawy A., Elhenawy Y. (2020). A novel concentrated photovoltaic-driven membrane distillation hybrid system for the simultaneous production of electricity and potable water. Renew. Energy.

[13] Mabrouk A.N., Abdelkader M., Shatat M. (2017). The impact of baffle orientation on the performance of the hollow fiber membrane. Desalin. Water Treat..

[14] Bassyouni M., Abdel-Aziz M.H., Zoromba M.S., Abdel-Hamid S., Drioli E. (2019). A review of polymeric nanocomposite membranes for water purification. J. Ind. Eng. Chem..

[15] Woo Y.C., Tijing L.D., Shim W.-G., Choi J.-S., Kim S.-H., He T., Drioli E., Shon H.K. (2016). Water desalination using graphene-enhanced electrospun nanofiber membrane via air gap membrane distillation. J. Membr. Sci..

[16] Alkhudhiri A., Darwish N.A., Hilal N. (2012). Membrane distillation: A comprehensive review. Desalination.

[17] Yang F., Efome J.E., Rana D., Matsuura T., Lan C. (2018). Metal–Organic Frameworks Supported on Nanofiber for Desalination by Direct Contact Membrane Distillation. ACS Appl. Mater. Interfaces.

[18] Lawal D.U., Khalifa A.E. (2014). Flux Prediction in Direct Contact Membrane Distillation. Int. J. Mater. Mech. Manuf..

[19] Elhady S., Bassyouni M., Mansour R.A., Elzahar M.H., Abdel-Hamid S.M.S., Elhenawy Y., Saleh M.Y. (2020). Oily Wastewater Treatment Using Polyamide Thin Film Composite Membrane Technology. Membranes.

[20] El-Marghany M.R., El-Shazly A.H., Salem M.S.A., Sabry M.N., Nady N. (2019). Novel Membrane Suitable for Membrane Distillation: Effect of Mixed Nanofillers on the Membrane Performance. Key Eng. Mater..

[21] Li X., Wang C., Yang Y., Wang X., Zhu M., Hsiao B.S. (2014). Dual-Biomimetic Superhydrophobic Electrospun Polystyrene Nanofibrous Membranes for Membrane Distillation. ACS Appl. Mater. Interfaces.

[22] Ihsanullah I. (2019). Carbon nanotube membranes for water purification: Developments, challenges, and prospects for the future. Sep. Purif. Technol..

[23] Baek Y., Kim H.J., Kim S.-H., Lee J.-C., Yoon J. (2017). Evaluation of carbon nanotube-polyamide thin-film nanocomposite reverse osmosis membrane: Surface properties, performance characteristics and fouling behavior. J. Ind. Eng. Chem..

[24] An A., Lee E.-J., Guo J., Jeong S., Lee J.-G., Ghaffour N. (2017). Enhanced vapor transport in membrane distillation via functionalized carbon nanotubes anchored into electrospun nanofibres. Sci. Rep..

[25] Silva T.L., Morales-Torres S., Figueiredo J.L., Silva A.M. (2015). Multi-walled carbon nanotube/PVDF blended membranes with sponge- and finger-like pores for direct contact membrane distillation. Desalination.

[26] Dumée L.F., Sears K., Schütz J., Finn N., Huynh C., Hawkins S., Duke M., Gray S.R. (2010). Characterization and evaluation of carbon nanotube Bucky-Paper membranes for direct contact membrane distillation. J. Membr. Sci..

[27] Tijing L.D., Woo Y.C., Shim W.-G., He T., Choi J.-S., Kim S.-H., Shon H.K. (2016). Superhydrophobic nanofiber membrane containing carbon nanotubes for high-performance direct contact membrane distillation. J. Membr. Sci..

[28] Zhou R., Rana D., Matsuura T., Lan C.Q. (2019). Effects of multi-walled carbon nanotubes (MWCNTs) and integrated MWCNTs/SiO_2_ nano-additives on PVDF polymeric membranes for vacuum membrane distillation. Sep. Purif. Technol..

[29] Ke H., Feldman E., Guzman P., Cole J., Wei Q., Chu B., Alkhudhiri A., Alrasheed R., Hsiao B.S. (2016). Electrospun polystyrene nanofibrous membranes for direct contact membrane distillation. J. Membr. Sci..

[30] Kulkarni A., Bambole V.A., Mahanwar P. (2010). Electrospinning of Polymers, Their Modeling and Applications. Polym. Technol. Eng..

[31] Nuraje N., Khan W.S., Lei Y., Ceylan M., Asmatulu R. (2013). Superhydrophobic electrospun nanofibers. J. Mater. Chem. A.

[32] Sadeghzadeh A., Bazgir S., Shirazi M.M.A. (2020). Fabrication and characterization of a novel hydrophobic polystyrene membrane using electroblowing technique for desalination by direct contact membrane distillation. Sep. Purif. Technol..

[33] Khoshnevisan S., Bazgir S. (2020). Treatment of dye wastewater by direct contact membrane distillation using superhydrophobic nanofibrous high-impact polystyrene membranes. Int. J. Environ. Sci. Technol..

[34] Parangusan H., Ponnamma D., Hassan M.K., Adham S., Al-Maadeed M.A. (2019). Designing Carbon Nanotube-Based Oil Absorbing Membranes from Gamma Irradiated and Electrospun Polystyrene Nanocomposites. Materials.

[35] Elmarghany M.R., El-Shazly A.H., Rajabzadeh S., Salem M.S.A., Shouman M.A., Sabry M.N., Matsuyama H., Nady N. (2020). Triple-Layer Nanocomposite Membrane Prepared by Electrospinning Based on Modified PES with Carbon Nanotubes for Membrane Distillation Applications. Membranes.

[36] Manawi Y., Wang K., Kochkodan V., Johnson D.J., Atieh M.A., Khraisheh M. (2018). Engineering the Surface and Mechanical Properties of Water Desalination Membranes Using Ultralong Carbon Nanotubes. Membranes.

[37] Sianipar M., Kim S.H., Khoiruddin K., Iskandar F., Wenten I.G. (2017). Functionalized carbon nanotube (CNT) membrane: Progress and challenges. RSC Adv..

[38] Esteves R.J.A., Gornick V., Alqurwani D.S., Koenig-Lovejoy J., Abdelrazeq H., Khraisheh M., Forzano A.V., Gad-El-Hak M., Tafreshi H.V., McLeskey J.T. (2020). Activated carbon-doped polystyrene fibers for direct contact membrane desalination. Emergent Mater..

[39] Amr I.T., Al-Amer A.M.J., Al-Harthi M.A., Girei S.A., Sougrat R., Atieh M.A. (2011). Effect of acid treated carbon nanotubes on mechanical, rheological and thermal properties of polystyrene nanocomposites. Compos. Part B Eng..

[40] Lehman J.H., Terrones M., Mansfield E., Hurst K.E., Meunier V. (2011). Evaluating the characteristics of multiwall carbon nanotubes. Carbon.

[41] León-Bermúdez Y., Salazar R. (2008). Synthesis and characterization of the polystyrene—Asphaltene graft copolymer by FT-IR spectroscopy. CT&F-Cienc. Tecnol. Futuro.

[42] Ding P., Qu B. (2006). Synthesis and characterization of polystyrene/layered double-hydroxide nanocomposites viain situ emulsion and suspension polymerization. J. Appl. Polym. Sci..

[43] Chamakh M.M., Ponnamma D., Al-Maadeed M.A.A. (2018). Vapor sensing performances of PVDF nanocomposites containing titanium dioxide nanotubes decorated multi-walled carbon nanotubes. J. Mater. Sci. Mater. Electron..

[44] Rabie M., Salem M.S., Ali A.Y., El-Shazly A., Elkady M., Ookawara S. (2020). Modeling of an integrated air gap membrane distillation unit utilizing a flat plate solar collector. Energy Rep..

[45] Hasanzadeh R., Darvishi M.M., Azdast T. (2019). Synergetic effect of MWCNT/nanoclays on microcellular polystyrene hybrid nanocomposite foams. Carbon Lett..

[46] Patole A.S., Patole S.P., Yoo J.B., An J.H., Kim T.H. (2012). Fabrication of Polystyrene/Multiwalled Carbon Nanotube Composite Films Synthesized by In Situ Microemulsion Polymerization. Polym. Eng. Sci..

[47] Karanasiou A., Kostoglou M., Karabelas A.J. (2018). An Experimental and Theoretical Study on Separations by Vacuum Membrane Distillation Employing Hollow-Fiber Modules. Water.

